# mito-TEMPO Attenuates Oxidative Stress and Mitochondrial Dysfunction in Noise-Induced Hearing Loss *via* Maintaining TFAM-mtDNA Interaction and Mitochondrial Biogenesis

**DOI:** 10.3389/fncel.2022.803718

**Published:** 2022-02-08

**Authors:** Jia-Wei Chen, Peng-Wei Ma, Hao Yuan, Wei-Long Wang, Pei-Heng Lu, Xue-Rui Ding, Yu-Qiang Lun, Qian Yang, Lian-Jun Lu

**Affiliations:** ^1^Department of Otolaryngology Head and Neck Surgery, Tangdu Hospital, Fourth Military Medical University, Xi’an, China; ^2^Department of Experimental Surgery, Tangdu Hospital, Fourth Military Medical University, Xi’an, China

**Keywords:** noise-induced hearing loss, reactive oxygen species, mitochondrial transcription factor A, mitochondrial biogenesis, sensory hair cells, rat model

## Abstract

The excessive generation of reactive oxygen species (ROS) and mitochondrial damage have been widely reported in noise-induced hearing loss (NIHL). However, the specific mechanism of noise-induced mitochondrial damage remains largely unclear. In this study, we showed that acoustic trauma caused oxidative damage to mitochondrial DNA (mtDNA), leading to the reduction of mtDNA content, mitochondrial gene expression and ATP level in rat cochleae. The expression level and mtDNA-binding function of mitochondrial transcription factor A (TFAM) were impaired following acoustic trauma without affecting the upstream PGC-1α and NRF-1. The mitochondria-target antioxidant mito-TEMPO (MT) was demonstrated to enter the inner ear after the systemic administration. MT treatment significantly alleviated noise-induced auditory threshold shifts 3d and 14d after noise exposure. Furthermore, MT significantly reduced outer hair cell (OHC) loss, cochlear ribbon synapse loss, and auditory nerve fiber (ANF) degeneration after the noise exposure. In addition, we found that MT treatment effectively attenuated noise-induced cochlear oxidative stress and mtDNA damage, as indicated by DHE, 4-HNE, and 8-OHdG. MT treatment also improved mitochondrial biogenesis, ATP generation, and TFAM-mtDNA interaction in the cochlea. These findings suggest that MT has protective effects against NIHL via maintaining TFAM-mtDNA interaction and mitochondrial biogenesis based on its ROS scavenging capacity.

## Introduction

Hearing loss is the most prevalent sensory disorder and affects more than 1.5 billion people worldwide (WHO, 2021). Common causes of acquired sensorineural hearing loss include exposure to loud noise (Sha and Schacht, 2017), aminoglycosides (He et al., 2017), platinum-based chemotherapy (Fernandez et al., 2020), heavy metals (Ding et al., 2019), and aging (He et al., 2021a). With the rapid development of modern society, noise has become one of the most common environmental pollutants in industrial and recreational settings. Exposure to high-intensity or chronic noise leads to noise-induced hearing loss (NIHL). Moderate noise leads to temporary threshold shift (TTS) that completely recovers to normal in 1–2 weeks. However, high-intensity or chronic noise can result in permanent threshold shift (PTS), i.e., irreversible hearing loss (Kurabi et al., 2017). According to WHO, about 10% of the world’s population is exposed to noise, 5.3% of whom develop NIHL (WHO, 2021). Those who experience hearing loss have difficulties in social communication, which may cause potential mental and cognitive disorders such as anxiety, depression, and dementia (Loughrey et al., 2018; Blazer and Tucci, 2019).

The cochlea is a metabolically active auditory sensory organ. Loud noise causes a dramatic increase in energy consumption of the cochlea (Chen et al., 2012). Production of reactive oxygen species (ROS) was observed in the cochlea in a few minutes following noise exposure, including superoxide (O_2_·-), hydroxyl radical (·OH), and hydrogen peroxide (H_2_O_2_). Moreover, ROS can be generated continuously for 7–10 days after cessation of the noise exposure (Ohlemiller et al., 1999; Yamashita et al., 2004). A lower level of ROS induced by moderate noise is reported to induce autophagy which protects against NIHL (Yuan et al., 2015). However, high-intensity noise induces excess ROS and activates 5’ adenosine monophosphate-activated protein kinase (AMPK), leading to hair cell death and hearing loss (Wu et al., 2020). Oxidative stress has been considered as a key factor in the mechanism of NIHL (Fetoni et al., 2019). ROS in the cochlea mainly comes from the mitochondrial metabolism of various cells. Mitochondrial pathology and dysfunction are associated with hearing loss of various etiologies (Fischel-Ghodsian et al., 2004; Böttger and Schacht, 2013). Mitochondria are essential organelles with several vital functions including energy production, maintenance of calcium homeostasis, and regulation of apoptosis (Lin and Beal, 2006). Due to the proximity to ROS production, mitochondria are also the first organelles damaged by ROS. The phenomenon of mitochondrial damage has been reported to be involved in NIHL in previous studies (Coleman et al., 2007; Park et al., 2012). However, the underlying mechanism has not been fully elucidated yet.

The mitochondrial DNA (mtDNA) is a circular double-stranded DNA located in the matrix of mitochondria, encoding 13 essential subunits of the mitochondrial respiratory chain (MRC; Scarpulla, 2008). Due to the lack of the repair mechanism and histone proteins like genomic DNA, mtDNA is more susceptible to the damage by ROS (Tsutsui et al., 2009). Mitochondrial transcription factor A (TFAM), a nucleus-encoded protein, is essential for the stabilization and maintenance of mtDNA. TFAM can bind to mtDNA in a sequence-independent manner to compact and stabilize the genome. In addition, TFAM can also bind sequence-specifically to promoter regions of mtDNA, initiating transcription and replication of mtDNA (Kang et al., 2007; Chandrasekaran et al., 2015). Previous studies have reported mitochondrial disintegration and vacuolization in hair cells (HCs) after acoustic trauma (Coleman et al., 2007; Park et al., 2012). However, the specific mechanism of noise-induced mitochondrial damage remains unclear. There are few studies on the alterations of the cochlear mtDNA content and expression level after acoustic trauma. Whether TFAM dysregulation is involved in the pathogenesis of NIHL is still unknown.

Recently, several novel antioxidants that specifically target mitochondria have been developed for the treatment of oxidative stress-related diseases (Zinovkin and Zamyatnin, 2019). 2-(2,2,6,6-tetramethylpiperidin-1-oxyl-4-ylamino)-2-oxoethyl (mito-TEMPO) is a mitochondria-target antioxidant with strong superoxide scavenging capacity and several hundred-fold accumulations in mitochondria (Shetty et al., 2019). A previous study demonstrated that mito-TEMPO (MT) can ameliorate renal fibrosis by reducing oxidative stress, mitochondrial dysfunction, and inflammation (Liu et al., 2018). In addition, recent studies have shown that MT has a protective effect against acetaminophen-induced hepatotoxicity with a wider therapeutic time window than N-acetyl-L-cysteine (NAC; Du et al., 2017; Abdullah-Al-Shoeb et al., 2020). Systemic administration of MT was able to alleviate ischemic brain damage in rats (Li et al., 2018). In addition, MT can pass through the blood-brain barrier (BBB) which is structurally similar to the blood-labyrinth barrier (BLB) in stria vascularis of the cochlea (Zhelev et al., 2013; Nyberg et al., 2019). However, whether MT has a protective effect on hearing has not been reported yet.

In the present study, we explored whether MT has a protective effect against NIHL and the underlying mechanism using an *in vivo* rat model. We first detected whether MT entered the inner ear after the systemic administration. Then we investigated the alleviation of noise-induced hearing loss, outer hair cell (OHC) loss, inner hair cell (IHC) ribbon synapse loss, and auditory nerve fiber (ANF) degeneration with systemic use of MT. We hypothesized that MT exerted the protective effect on hearing via reducing mtDNA oxidative damage, stabilizing the TFAM-mtDNA interaction, maintaining mitochondrial biogenesis and function.

## Materials and Methods

### Animals

Sixty-two female adult Sprague-Dawley rats (200–250 g) were used in this study. Two rats were used for the determination of MT in the cochlea. Sixty Rats were randomly assigned to three groups (*n* = 20 for each group): control, noise + vehicle treatment, noise + MT treatment. All animals were kept at 22 ± 1°C under a 12 h light/12 h dark cycle with free access to water and food. All procedures of the animal experiments were approved by the Institutional Animal Care and Use Committee of Fourth Military Medical University.

### Auditory Brainstem Response (ABR) Measurements

The hearing function of 18 animals (*n* = 6 for each group) was evaluated by auditory brainstem response (ABR) at four frequencies of 8, 16, 24, and 32 kHz. ABR measurements were carried out 2 days before (−2d), 3 days after (+3d) and 14 days after (+14d) the noise exposure to evaluate the baseline hearing function and noise-induced auditory threshold shifts. Rats were anesthetized with an intraperitoneal injection of the cocktail anesthetic (chloral hydrate, pentobarbital sodium, and magnesium sulfate). Body temperature was maintained at near 37°C with a heating pad. Three subdermal electrodes were inserted at the vertex of the skull (active), the left mastoid (reference), and the right mastoid (ground). Tucker-Davis Technologies (TDT) RZ6 System was used to generate acoustic stimuli and record the responses. Tone pip stimuli at 8, 16, 24, and 32 kHz (5-ms-duration with 2.5-ms rise–fall time) were delivered monaurally with an earphone inserted into the external auditory canal of the left ear. Acoustic stimuli were generated from 90 dB and then attenuated in 10-dB and 5-dB steps until no ABR waves were recognizable. A total of 1,024 responses were averaged for each stimulus level. ABR wave II was used to determine ABR thresholds for each frequency. The thresholds were defined as the lowest intensity able to evoke repeatable response waves.

### Drug Administration

MT (Sigma-Aldrich, SML0737) was dissolved in normal saline as a stock solution and stored at −20°C. The stock solution was diluted with normal saline at a concentration of 0.5 mg/ml before the injection. For animals in the MT treatment group, a dose of 1 mg/kg was selected based on the previous studies (Liu et al., 2018; Xing et al., 2021). For the animals to be sacrificed on day 1, MT was injected intraperitoneally (i.p.) 24 h before, 1 h before, and immediately after noise exposure. The Animals to be sacrificed on day 3 and 14 received three additional injections once daily for the following 3 days. The animals in the control group and the vehicle treatment group received three or six injections of normal saline with the same experimental procedure.

### Perilymph Extraction and Sample Preparation

Two rats were injected intraperitoneally with 1 mg/kg MT. Fifteen minutes after the injection, rats were sacrificed and the cochleae were harvested. Then 2 μl of perilymph was sampled using a capillary tube from the round window of the cochlea. A total of 8 μl of perilymph was pooled and used for the determination of MT. Then 100 μl of methanol was added to the sample. After vortexing, the sample was centrifuged at 5,000×g for 10 min at 4°C. The supernatant was retained and used for liquid chromatography tandem mass spectrometry (LC-MS/MS) analysis.

### Chromatographic Separation and MS/MS Detection

The LC-MS/MS analysis was performed with the Triple Quadrupole LC/MS (Agilent, 6470B, USA). Chromatographic separation was performed using an Agilent C18 column (100 × 2.1 mm i.d., 3 μm particle size). The sample injection volume was 10 μl. The column temperature was maintained at 35°C. Then a gradient of mobile phase A (0.1%(v/v) formic acid in 5 mM ammonium acetate) and mobile phase B (acetonitrile) was run at 0.3 ml/min as follows: 95% A and 5% B, 0–15 min; 20% A and 80% B, 15–17 min; 100% A, 17–20 min. The MS/MS analysis was performed in the positive electrospray ionization (ESI+) mode. The source temperature was kept at 500°C. The ion spray voltage was 4,500 V, and the curtain gas was set to 35 psi. The detection of the ions was operated in the multiple reaction monitoring (MRM) mode, monitoring transitions of m/z 474.2 → 320.1 for MT. The quantification of the MT in the perilymph was analyzed by calculating the peak area ratios using the external standard.

### Acoustic Trauma

The acoustic trauma was induced by continuous noise exposure. The rats were placed separately in wire cages located in a sound-proof room. The acoustic signal of 8–16 kHz octave band noise was generated by RZ6 Noise software. Then the signal was amplified and output by the power amplifier (Crown, XLi800, China) and four loudspeakers (CHUANGMU, CP-75A, China). The level of the noise was maintained at 112 ± 1 dB SPL confirmed by the Sound Level Meter. Animals were exposed to noise for 2 h.

### Immunofluorescence Histochemistry for Surface Preparations and Frozen Sections of the Cochlea

Twelve animals used for the analyses of 4-HNE, DHE, 8-OHdG level, and TFAM-mtDNA interaction were sacrificed 1 h after the noise exposure. Eighteen animals used for analyses of OHC loss, IHC ribbon synapse loss, and ANF degeneration were sacrificed 14d after the noise exposure. Rat cochleae were quickly dissected from the temporal bones following rapid decapitation. The round and oval windows were opened after removing the stapes. Then the cochleae were gently perfused with 4% paraformaldehyde (PFA) in 0.1 M phosphate-buffered saline (PBS) via the round window, and kept in the fixative overnight at 4°C. After the fixation, the cochleae were rinsed three times with PBS for 5 min and then decalcified with 10% ethylenediaminetetraacetic acid (EDTA) for 5 days at room temperature with a daily solution change. After the decalcification, the cochleae were rinsed three times with PBS for 5 min. For cochlear surface preparations, the organ of Corti (OC) was dissected in PBS by carefully removing the lateral wall, Reissner’s membrane, and the tectorial membrane of the cochlea using micro-dissecting forceps and scissors under a stereomicroscope (Olympus, SZ61, Japan). Then the specimens were transferred into a 96-well plate, followed by permeabilization using 1% Triton X-100 in PBS for 15 min at room temperature. The specimens were blocked with immunol staining blocking buffer (Leagene, IH0338) for 1 h at room temperature. Then the specimens were incubated overnight at 4°C with the following primary antibodies: Myosin VIIa (Proteus Biosciences, #25-6790, 1:1,000), TFAM (Abcam, ab252432, 1:500), dsDNA (Abcam, ab27156, 1:500). After being rinsed with PBS three times, the specimens were incubated for 2 h at room temperature in darkness with following secondary antibodies: donkey anti-rabbit IgG Alexa Fluor 594 (Invitrogen, A21207, 1:200), donkey anti-mouse IgG Alexa Fluor 488 (Invitrogen, A21202, 1:200). After being rinsed with PBS three times, the specimens were incubated with Acti-Stain 670 Phalloidin (Cytoskeleton, #PHDN1-A, 1:70) or DAPI (Roche, #10236276001, 1 μg/ml) for 30 min at room temperature.

For immunolabeling of IHC ribbon synapses, the specimens were permeabilized with 3% Triton X-100 in PBS for 30 min at room temperature. After blocking for 1 h, the specimens were incubated overnight at 37°C with following primary antibodies: Myosin VIIa (Proteus Biosciences, #25-6790, 1:1,000), CtBP2 (BD Bioscience, #612044, 1:500), GluR2 (Alomone Labs, AGC-005, 1:1,000). After being rinsed with PBS three times, the specimens were incubated for 2 h at room temperature in darkness with following secondary antibodies: goat anti-rabbit IgG Alexa Fluor 568 (Invitrogen, A11036, 1:200), goat anti-mouse IgG1 Alexa Fluor 647 (Invitrogen, A21240, 1:200), goat anti-mouse IgG2a Alexa Fluor 488 (Invitrogen, A21131, 1:200). After the final rinse with PBS, each specimen was dissected into the apex, middle and base turns. Each cochlear turn was transferred to the slide and placed in the mounting medium (Vector, H-1000). Finally, the specimen was covered with a coverslip and the edge was sealed with transparent nail polish.

For the preparation of cochlear frozen sections, the decalcified cochleae were incubated in 10% sucrose and 20% sucrose for 4 h and 30% sucrose overnight at 4°C for dehydration. Then the specimens were embedded in an optimal cutting temperature (OCT) compound. The midmodiolar cryosections at a thickness of 8 μm were prepared using a Cryostat Microtome (Leica, CM1860, Germany). The sections were permeabilized with 0.5% Triton X-100 in PBS for 15 min at room temperature, followed by blocking with immunol staining blocking buffer for 1 h at room temperature. Then the sections were incubated overnight at 4°C with following primary antibodies: Myosin VIIa (Proteus Biosciences, #25-6790, 1:1,000), Tuj1 (GeneTex, GTX631836, 1:200), 4-HNE (GeneTex, GTX17571, 1:50), 8-OHdG (Abcam, ab48508, 1:200), Tuj1(ABclonal, A17913, 1:500). After being rinsed with PBS three times, the specimens were incubated with secondary antibodies for 2 h at room temperature in darkness. For DHE staining, the permeabilized sections were incubated with 1 μM DHE solution (Beyotime, S0063) for 30 min at 37°C in darkness. After being rinsed with PBS three times, a drop of mounting medium with DAPI (Vector, H-1200) was added onto the section. Finally, the section was covered with a coverslip and the edge was sealed with transparent nail polish. Images were obtained with the Confocal Laser Scanning Microscope (CLSM; Olympus, FV1000, Japan) under identical parameter settings in each experiment.

### Quantitative or Semi-quantitative Analyses of the Fluorescence Signals

The quantitative or semi-quantitative analyses of the fluorescence signals were conducted with ImageJ software (version 1.53a, USA). For the OHC counting 14d after noise exposure, cochlear surface preparations labeled with Myosin VIIa and DAPI were used. The OHC counting was measured in apex, middle and base turns of surface preparations in 0.3-mm segments. The absence of both the nucleus (DAPI) and the cell body (Myosin VIIa) was considered as the OHC loss. The mean OHC survival in each group was calculated with four specimens. The quantification of IHC ribbon synapses was analyzed in the 0.1-mm segment of the surface preparation (containing about 10 IHCs), corresponding to frequencies of 16–20 kHz. The juxtaposition of CtBP2 and GluA2 was considered as the paired synapse. The total number of paired fluorescent spots was counted and divided by the number of IHC within the image. For the analysis of ANF density, cochlear frozen sections immunolabelled with Tuj1 and Myosin VIIa were used. The intensity of Tuj1 fluorescent signals in nerve fibers in the upper-base turn of the cochlea was analyzed and considered as ANF density. For the semi-quantitative analyses of 8-OHdG and 4-HNE expression, the intensity of the fluorescent signals in SGNs, HCs, or SV region in the upper-base turn of the cochlea was analyzed. Briefly, the images were converted into 8-bit grayscale type. The intensity of the background signal was subtracted and the average grayscale intensity of SGNs, HCs or SV region was then measured. The relative grayscale values were calculated by normalizing the ratio to the control group. The quantification of TFAM-mtDNA interaction was analyzed in the 70-μm segment at the base turn of the surface preparation. Those green fluorescent spots (dsDNA) in the cytoplasm that were not colocalized with red ones (TFAM) were considered as naked mtDNA. The total naked mtDNA spots in OHCs were counted and divided by the number of OHC within the image. All quantitative and semi-quantitative analyses were performed in four specimens for each group.

### Extraction of Cochlear DNA and RNA

Six animals were sacrificed on day 1 after the third injection and six animals were sacrificed on day 3 after the ABR testing. The cochleae were dissected in Hanks’ balanced salt solution on ice and frozen in liquid nitrogen until use. Twelve cochleae (*n* = 4 for each group) of the animals sacrificed on day 1 were used for the extraction of total RNA. Twelve cochleae (*n* = 4 for each group) of the animals sacrificed on day 3 were used for genomic DNA (gDNA) extraction. The gDNA and total RNA were extracted using the Tissue DNA Extraction Kit (TIANGEN, DP304) and RNeasy Plus Mini Kit (QIAGEN, #74134) according to the manufacturer’s instructions, respectively. The purity and concentration of the DNA and RNA products were analyzed using the microplate spectrophotometer (BioTek, Epoch, USA). Then the isolated RNA was reverse transcribed to cDNA using PrimeScript RT Master Mix (TaKaRa, RR036A). The gDNA and cDNA products were stored at −20°C until use.

### Quantification of mtDNA Copy Number and Mitochondrial Nd6 mRNA Expression

The mtDNA copy number and mitochondrial Nd6 expression were quantified by real-time polymerase chain reaction (PCR) assay using Real-Time PCR Detection System (Bio-Rad, CFX96, USA). The real-time PCR reaction system was prepared using TB Green Premix Ex Taq II (TaKaRa, RR820A) according to the manufacturer’s instructions. The nuclear gene β-actin was used as the internal control. For the quantification of mtDNA copy number, the gDNA was used for the template. The cycle threshold (Ct) values of 12S rRNA (mtDNA) and β-actin (nDNA) were used to determine the relative mtDNA copy number. The following primers were designed: β-actin Forward: CTACCTCGCTGCAGGATCG, β-actin Reverse: GTCTACACCGCGGGAATACG, 12S rRNA Forward: AAGGAGAGGGCATCAAGCAC, and 12S rRNA Reverse: TATCACTGCTGAGTCCCGTG.

For the measurement of mitochondrial Nd6 mRNA expression, the cDNA was used as a template. The relative mRNA expression was estimated by the Ct values of Nd6 (mtDNA expression) and β-actin (nDNA expression). The following primers were designed: β-actin Forward: GGAGATTACTGCCCTGGCTCCTA, β-actin Reverse: GACTCATCGTACTCCTGCTTGCTG, Nd6 Forward: ACCCTCAAGTCTCCGGGTA, and Nd6 Reverse: GTCTAGGGTTGGCGTTGAAG. The relative levels of mtDNA copy number and mRNA expression in each group were analyzed using the 2^−ΔΔCt^ method.

### ATP Level Measurement

Twelve animals (*n* = 4 for each group) in each group were sacrificed for cochlear ATP level measurement 1 h after the noise exposure. Two cochleae of an animal were quickly harvested and pooled as one sample. ATP levels in the cochlea were measured based on spectrophotometry using ATP Detection Kit (Solarbio, BC0305) according to the manufacturer’s instructions. The working principle of the Kit is briefly described as follows. The Glucose and ATP are catalyzed by hexokinase to produce glucose 6-phosphate, which is further catalytically dehydrogenated to produce NADPH. The NADPH shows a characteristic absorption peak at 340 nm. The content of ATP is proportional to that of NADPH.

### Extraction of Cochlear Protein and Immunoblotting

Twelve cochleae (*n* = 4 for each group) of six animals sacrificed on day 1 were homogenized in ice-cold RIPA lysis buffer (Beyotime, P0013B) containing protease inhibitor cocktail (Roche, #04693159001, Switzerland) using the cryogenic grinder. The tissue homogenate was placed on ice for 30 min, and then centrifuged at 12,000× *g* for 10 min at 4°C. The supernatant was retained and the protein concentration was determined using the BCA Determination Kit. Then the supernatant added with loading buffer was boiled for 5 min and stored at −20°C.

The protein samples (30 μg) were separated by 10–12% sodium dodecyl sulfate polyacrylamide gel electrophoresis (SDS-PAGE) electrophoresis and transferred to polyvinylidene fluoride (PVDF) membranes (Millipore, IPVH00010). After being blocked with 5% skimmed milk in TBS-T, the membranes were incubated for 1 h at room temperature on shaker and then overnight at 4°C with following primary antibodies: PGC-1α (Abcam, ab191838, 1:1,000), NRF-1 (GeneTex, GTX103179, 1:1,000), TFAM (Abcam, ab131607, 1:1,000), SOD2(GeneTex, GTX116093, 1:1,000), Bax (GeneTex, GTX109683, 1:1,000), ACTB (GeneTex, GTX109639, 1:2,000). The membranes were rinsed three times with TBS-T for 5 min, followed by incubation with HRP-linked secondary antibody (CST, #7074, 1:2,000) for 1 h at room temperature on a shaker. After being rinsed with TBS-T three times, the blots were visualized with chemiluminescent HRP substrate (Millipore, WBKLS0100) and scanned using the Chemiluminescence Imaging System (Fusion, Solo 6S, China). The band intensities were quantified using the ImageJ software (version 1.53a, USA) and normalized by using ACTB as the internal control.

### Statistics

Statistical analyses were conducted using IBM SPSS software (version 25.0, USA) and GraphPad Prism software (version 8.0, USA). The group comparisons were performed using two-tailed, two-sample Student’s *t*-test or one-way analysis of variance (ANOVA). When the difference was significant, the LSD *post hoc* test was used to identify the difference between the two groups. The *p*-value of <0.05 was considered statistical significance. All the data were presented as mean ± standard deviation (SD).

## Results

### MT Entered Inner Ear After Systemic Administration

In order to investigate whether MT existed in the inner ear after the systemic administration, we analyzed the concentration of MT in the perilymph of the cochlea using LC-MS/MS. The perilymph was extracted 15 min after the injection and used for the detection and quantification of MT. As shown in Supplementary Figures 1A–C, the retention time of the MT in the perilymph was 3.302 min. The mass-to-charge ratio (m/z) of the parent ion was 474.2, which was consistent with the relative molecular mass of MT losing a chloride ion (Supplementary Figure 1D). The concentration of the MT in the perilymph of the cochlea was 18.541 μg/kg 15 min after the injection. These results indicated that MT could pass through the BLB and enter the inner ear.

### Administration of MT Attenuated Noise-Induced Auditory Threshold Shift

**Figure 1:**
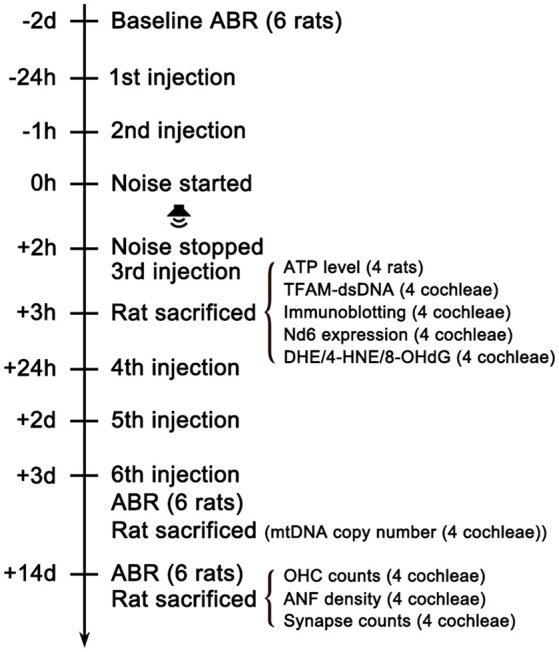
Experimental timeline. The baseline hearing function of the rats was evaluated via ABR testing 2 days (2d) before the noise exposure. Rats were intraperitoneally injected with MT or vehicle (saline) 24 h and 1 h before the noise started. Then rats were exposed to 112dB SPL 8–16 kHz octave band noise for 2 h. The third shot was injected immediately after the noise stopped. One hour later, 12 animals in each group were sacrificed for analyses of oxidative stress level, mtDNA expression quantification, ATP measurement, immunoblotting and TFAM-mtDNA interaction. The rest of the animals received three additional injections once daily for the following 3 days. Then ABR testing were performed 3d after the noise exposure to evaluate TTS level. Then two animals in each group were sacrificed for the quantification of cochlear mtDNA copy number. After ABR testing 14d after the noise exposure for PTS level assessment, the remaining animals were sacrificed for analyses of OHC loss, IHC ribbon synapse loss and ANF degeneration. ABR, Auditory Brainstem Response.

**Figure 2:**
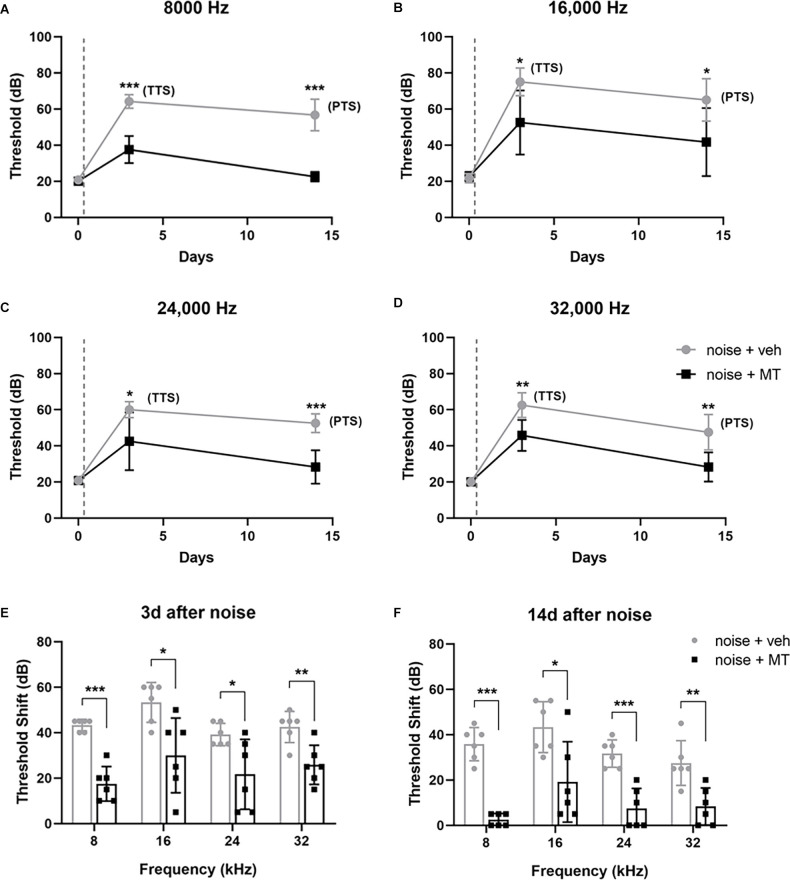
MT treatment attenuated noise-induced auditory threshold shifts. **(A–D)** The auditory threshold alterations of the left ear of animals at 8, 16, 24 and 32 kHz over time. The baseline threshold, TTS, and PTS were evaluated before, 3d after and 14d after the noise exposure respectively. **(E,F)** The auditory threshold shifts over four frequencies on the day 3 and the day 14. The gray dotted lines indicate the time of acoustic trauma. Data are presented as means ± SD, *n* = 6 for each group, *p < 0.05, **p < 0.01, ***p < 0.001. MT, mito-TEMPO; TTS, temporary threshold shift; PTS, permanent threshold shift.

To address whether systemic use of MT might prevent NIHL, we designed the experimental procedure as shown in Figure 1. The hearing function of the animals was evaluated via ABR testing 2 days before (−2d), 3 and 14 days after (+3d and +14d) noise exposure. There is no significant difference in baseline ABR threshold between groups (Supplementary Figure 2). Compared with saline-treated rats, MT-treated rats showed significantly decreased TTS on day 3 and PTS on day 14 at 8, 16, 24, and 32 kHz (Figures 2A–D). The 112dB noise exposure for 2 h resulted in about 30–40 dB threshold shift at all four frequencies on day 14 in the vehicle-treated group. However, in the MT-treated group, the threshold at 8 kHz was completely recovered and threshold shifts at 24 and 32 kHz were both within 10 dB on day 14 (Figures 2E,F). These data indicated that MT administration was able to attenuate noise-induced transient and permanent hearing loss.

### MT Treatment Prevented OHC Loss Induced by Acoustic Trauma

**Figure 3:**
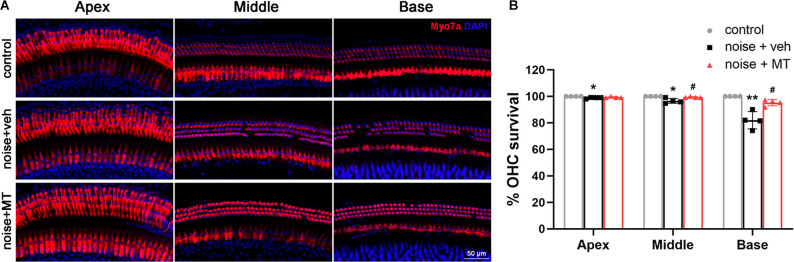
MT treatment prevented noise-induced OHC loss. **(A)** Representative images of the apex, middle and base turn region of cochlear surface preparations 14 days after the noise exposure. HCs were indicated by Myosin VIIa (red) and DAPI (blue) fluorescent staining. **(B)** Quantification analysis of OHC survival in the apex, middle and base turn region of the cochlea 14 days after the noise exposure. Data are presented as means ± SD, *n* = 4 for each group, *p < 0.05 vs. control group, **p < 0.01 vs. control group, ^#^*p* < 0.05 vs. vehicle-treated group. OHC, outer hair cell; HCs, hair cells.

We then asked if MT exhibits a protective effect on the OHC, which is one of the most vulnerable structures of the cochlea during acoustic trauma. We used the surface preparation technique to analyze the OHC loss 14 days after the noise exposure. The cochlea was divided into the apex, middle and base turns, which correspond to the low-, middle- and high-frequency regions of the cochlea. The loss of both the nucleus (DAPI) and the cell body (Myosin VIIa) was considered as the OHC loss. As shown in Figures 3A,B, noise overstimulation resulted in the most OHC loss at the base turn in the vehicle-treated group, showing 81.5% of the OHC survival. Compared with the vehicle-treated group, MT treatment significantly reduced noise-induced OHC loss at the base and middle turns of the cochlea. The OHC survival reached 95.2% at the base turn in the MT-treated group. These results indicated that MT was able to prevent OHC death after acoustic trauma.

### MT Treatment Reduced IHC Ribbon Synapse Loss and ANF Degeneration Induced by Acoustic Trauma

**Figure 4:**
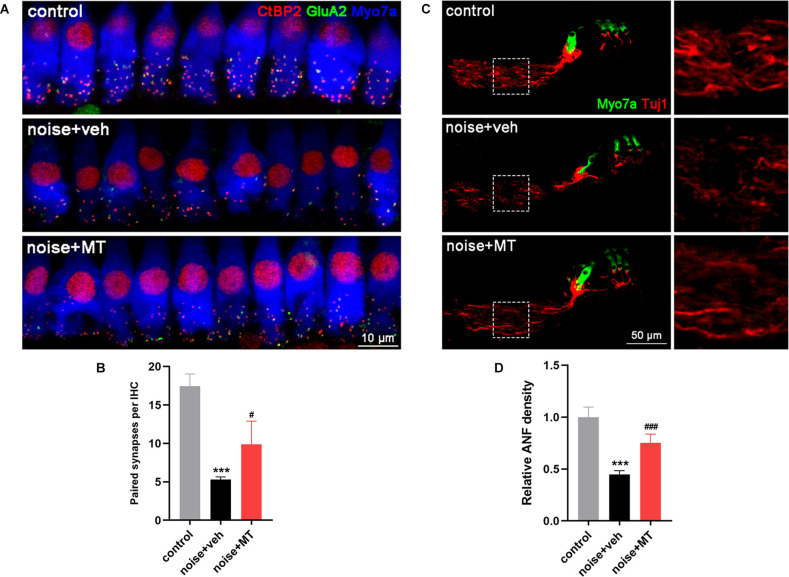
MT treatment reduced IHC ribbon synapse loss and ANF degeneration induced by acoustic trauma. **(A)** Representative images of the IHC ribbon synapse at approximately 16–20 kHz region of the cochlea 14d after the noise exposure. Cochlear surface preparations were immunolabelled with CtBP2 (red), GluA2 (green) and Myosin VIIa (blue). **(B)** Quantification analysis of paired synapse per IHC, indicated by the juxtaposition of CtBP2 and GluA2. **(C)** Representative images of ANF and HCs in middle turn area of the midmodiolar sections 14d after the noise exposure. Cochlear sections were immunolabelled with Tuj1 (red) and Myosin VIIa (green). **(D)** Quantification analysis of ANF density 14d after the noise exposure. Data are presented as means ± SD, *n* = 4 for each group, ***p < 0.001 vs. control group, ^#^*p* < 0.05 vs. vehicle-treated group, ^###^*p* < 0.001 vs. vehicle-treated group. IHC, inner hair cell; ANF, auditory nerve fiber.

Recent studies show that the loss of ribbon synapses between IHCs and spiral ganglion neurons (SGNs) is the primary pathology in NIHL, which is termed cochlear synaptopathy (Liberman and Kujawa, 2017). To explore the protective effect of MT against noise-induced IHC ribbon synapse loss, we quantified the number of paired ribbon synapses 14 days after the noise exposure. The surface preparations of the cochlea at approximately 16–20 kHz region were immunolabelled with antibodies against CtBP2 and GluA2 to manifest the pre- and post-synaptic structures. The juxtaposition of CtBP2 and GluA2 represents the functional synapses. In the vehicle-treated group, a significant decrease in the number of paired synapses was observed after noise exposure. Compared with the vehicle-treated group, treatment with MT significantly reduced the noise-induced loss of the paired ribbon synapses (Figures 4A,B).

In addition, NIHL is associated with the degeneration of ANFs which extend from SGNs to IHC ribbon synapses (Kujawa and Liberman, 2009). We next explored the protective effect of MT against noise-induced ANF degeneration by measuring the density of the fiber 14 days after the noise exposure. Compared with the control group, there was more than 50% degeneration of ANF in the vehicle-treated group. The remaining nerve fibers were fragmented and in a disordered arrangement. However, treatment with MT significantly recovered the density of ANF after acoustic trauma. The integrity of nerve fibers was partially restored in the MT-treated group (Figures 4C,D). These results indicated the neuroprotective effect of the MT against IHC ribbon synapse loss and ANF degeneration in NIHL.

### MT Treatment Mitigated Oxidative Stress in the Cochlea After the Noise Exposure

**Figure 5:**
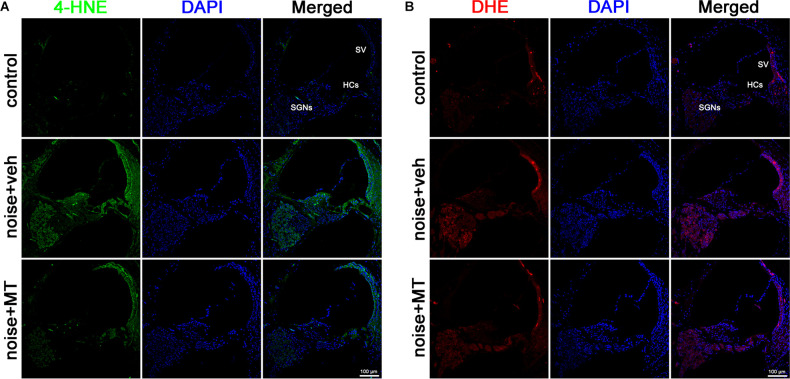
MT treatment mitigated ROS generation in the cochlea. **(A)** Representative images of 4-HNE (green) and DAPI (blue) fluorescent staining in base turn area of the midmodiolar sections 1 h after the noise exposure. **(B)** Representative images of DHE (red) and DAPI (blue) fluorescent staining in base turn area of the midmodiolar sections 1 h after the noise exposure. Four cochleae form two animals were used in each group. HCs, hair cells; SV, stria vascularis; SGNs, spiral ganglion neurons; ROS, reactive oxygen species.

Excessive ROS production is a major causative factor in noise-induced cochlear injury and hearing loss. ROS oxidizes polyunsaturated fatty acids to produce cytotoxic aldehydes. Among these, 4-hydroxynonenal (4-HNE) represents one of the major products of lipid peroxidation (Di Domenico et al., 2017). Dihydroethidium (DHE) is a fluorescent dye for detecting the level of superoxide ion (O_2_·-). In the presence of O_2_·-, DHE is oxidized to 2-hydroxyethidium, the fluorescence of which can be measured at an excitation and emission wavelength of 480 nm and 567 nm (Fetoni et al., 2015). In the present study, the level of cochlear oxidative stress was analyzed by 4-HNE and DHE fluorescent staining. As shown in Figure 5A, noise overstimulation induced a substantial increase of 4-HNE generation in the cochlea. Increased 4-HNE was mainly observed in HCs, stria vascularis (SV), and SGNs of the cochlea (Supplementary Figure 3). Compared with the vehicle-treated group, MT treatment significantly attenuated 4-HNE generation after the noise exposure. The DHE assay showed similar results. MT treatment remarkably reduced the DHE fluorescence induced by the noise overstimulation (Figure 5B). These data indicated that MT alleviated noise-induced oxidative stress in the cochlea by reducing the level of lipid peroxidation and O_2_·- generation.

### MT Treatment Alleviated mtDNA Oxidative Damage, and Maintained Mitochondrial Biogenesis and ATP Production After the Acoustic Trauma

**Figure 6:**
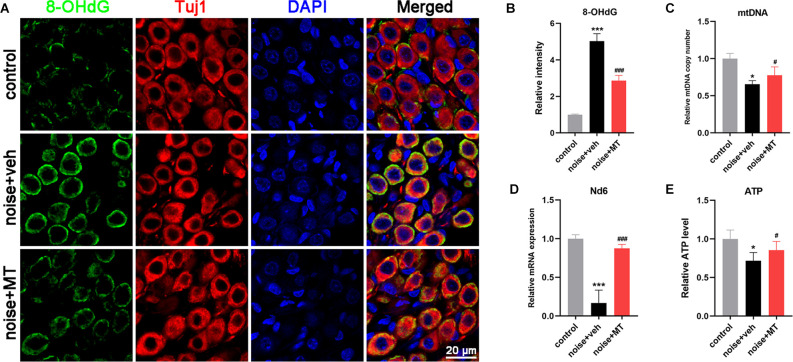
MT treatment alleviated mtDNA oxidative damage, and maintained mitochondrial biogenesis and ATP production after the acoustic trauma. **(A)** Representative images of 8-OHdG (green), Tuj1 (red) and DAPI (blue) fluorescent staining in SGNs in frozen sections of the cochlea 1 h after the noise exposure. **(B)** Semi-quantitative analysis of the fluorescent intensity of 8-OHdG in SGNs 1 h after the noise exposure. **(C)** Real time-qPCR analysis of mtDNA copy number in the cochlea 3d after the noise exposure. **(D)** Real time-qPCR analysis of mitochondrial Nd6 expression level in the cochlear 1 h after the noise exposure. **(E)** The ATP level in the cochlea 1 h after the noise exposure. Data are presented as means ± SD, *n* = 4 for each group, *p < 0.05 vs. control group, ***p < 0.001 vs. control group, ^#^*p* < 0.05 vs. vehicle-treated group, ^###^*p* < 0.001 vs. vehicle-treated group.

The mitochondria are semi-autonomous organelles that contain their own circular genetic system. The mitochondrial genomes encode 13 essential protein subunits of the MRC complexes. As is located close to the site of ROS generation, mtDNA molecules are easily damaged, resulting in decreased mtDNA content and mitochondrial dysfunction (Kang et al., 2007). 8-hydroxy-2’-deoxyguanosine (8-OHdG) is one of the most abundant oxidative adducts of DNA, reflecting the level of DNA oxidative damage (Han et al., 2020). In Figures 6A,B, a dramatic increase of 8-OHdG production was observed in the cytoplasm of SGNs following noise exposure. Since the DNA in cytoplasm refers to mtDNA, increased cytoplasmic 8-OHdG indicated that acoustic trauma-induced mtDNA oxidative damage in SGNs. Compared with the vehicle-treated group, treatment of MT significantly reduced noise-induced 8-OHdG production in SGNs.

Mitochondrial biogenesis relies on mtDNA replication and transcription to maintain mtDNA content and expression, and to produce new mitochondria. mtDNA is a multicopy genome and maintained at a relatively stable content (Scarpulla, 2008). Noise exposure resulted in a significant reduction of mtDNA content in the cochlea, reflected by the mtDNA copy number (Figure 6C). The mRNA level of MRC gene mt-Nd6 was also decreased after noise exposure, suggesting that the mtDNA expression in the cochlea was impaired by acoustic trauma (Figure 6D). In addition, ATP level in the cochlea was decreased following noise exposure, which indicated the dysfunction of the MRC (Figure 6E). However, treatment with MT significantly restored mtDNA content, mtDNA expression, and ATP level in the cochlea after noise exposure compared with the vehicle-treated group. These data indicated that MT was able to alleviate noise-induced mtDNA oxidative damage, maintain mitochondrial biogenesis and improve mitochondrial function of the cochlea.

### MT Treatment Mitigated Noise-Induced Downregulation of TFAM and SOD2 Independent of the PGC-1α/NRF-1/TFAM Pathway

**Figure 7:**
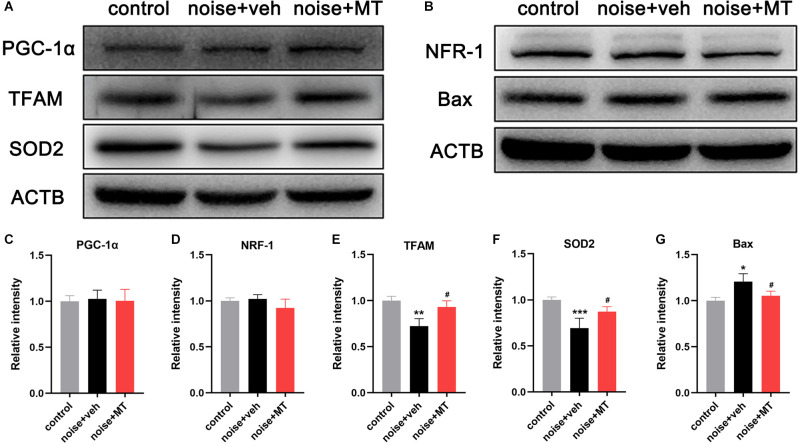
MT treatment mitigated noise-induced downregulation of TFAM and SOD2 independent of the PGC-1α/NRF-1/TFAM pathway. **(A,B)** Immunoblotting of the PGC-1α, NRF-1, TFAM, SOD2, Bax, and ACTB protein in the cochlea 1 h after the noise exposure. **(C–G)** Quantification analysis of the optical density values of the immunoblot bands normalized by using ACTB as the internal control. Data are presented as means ± SD, *n* = 4 for each group, *p < 0.05 vs. control group, **p < 0.01 vs. control group, ***p < 0.001 vs. control group, ^#^*p* < 0.05 vs. vehicle-treated group.

Mitochondrial biogenesis is a dynamic process of the generation of new mitochondria by synthesizing mitochondrial DNA and proteins. This activity requires several nucleus-encoded transcription factors, including peroxisome proliferator-activated receptor-gamma coactivator-1alpha (PGC-1α), nuclear respiratory factor 1 and 2 (NRF-1 and NRF-2), mitochondrial transcription factor A (TFAM), B1 (TFB1M), and B2 (TFB2M; Scarpulla, 2008). The PGC-1α/NRF-1/TFAM axis is the most studied pathway in regulating the replication and expression of mtDNA and mitochondrial biogenesis (Zhao et al., 2013; Xiong et al., 2019). In the above experiment, we found that acoustic trauma led to the decline of mtDNA content and expression level. To further explore the underlying mechanism, we next investigated the expression of PGC-1α/NRF-1/TFAM after noise exposure. As shown in Figures 7A–D, the expression level of PGC-1α and NRF-1 did not change following noise exposure. However, noise overstimulation resulted in a significant decrease in TFAM expression in the cochlea (Figures 7A,E). These results indicated that noise-induced decline of mtDNA content and expression was associated with TFAM reduction independent of PGC-1α/NRF-1/TFAM pathway. Compared with the vehicle-treated group, MT treatment attenuated TFAM expression reduction after the noise exposure.

Of the family of superoxide dismutase (SODs), only SOD2 (Mn-SOD) is located in mitochondria (Gross et al., 2014). We observed that noise exposure induced a dramatic decrease in the expression of mitochondrial SOD2 (Figures 7A,F). MT treatment partially improved SOD2 expression compared with the vehicle-treated group. In addition, the expression of the pro-apoptotic protein Bax was slightly upregulated following acoustic trauma and alleviated by MT treatment (Figures 7B,G). These data suggested that MT prevented noise-induced disruption of the mitochondrial antioxidant defense, and inhibited apoptosis activation in the inner ear.

### MT Treatment Improved the Disruption of TFAM-mtDNA Interaction in the Cochlea After the Noise Exposure

**Figure 8:**
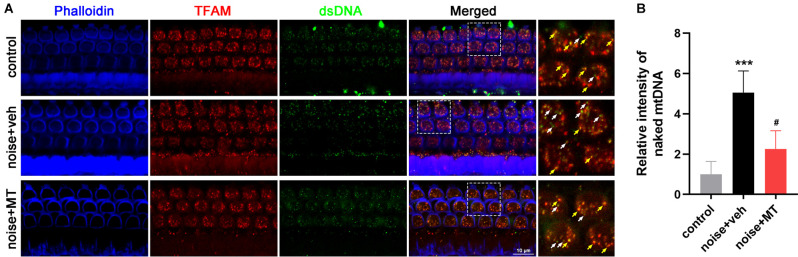
MT treatment improved the disruption of TFAM-mtDNA interaction in the cochlea after the noise exposure. **(A)** Representative images of Phalloidin (blue), TFAM (red), and dsDNA (green) fluorescent staining in base turn area of the cochlear surface preparation 1 h after the noise exposure. Colocalized spots of TFAM and dsDNA were indicated in yellow arrows, while “orphan” dsDNA spots without colocalization of TFAM were indicated in the white arrows. **(B)** Quantification analysis of naked mtDNA in OHCs 1 h after the noise exposure. Data are presented as means ± SD, *n* = 4 for each group, ***p < 0.001 vs. control group, ^#^*p* < 0.05 vs. vehicle-treated group.

TFAM binds the D-loop region of the mitochondrial genome to initiate mtDNA transcription and replication. TFAM also maintains the stability of mtDNA in a sequence-independent binding manner. We investigated the mtDNA-binding ability of TFAM by immunofluorescence colocalization analysis of TFAM and the double-stranded DNA (dsDNA) in the cytoplasm. As shown in Figure 8A, most mtDNA molecules were bound to TFAM (yellow arrows) within sensory hair cells labeled by Phalloidin in the control group. However, noise exposure induced a reduction of TFAM-mtDNA interaction, leading to a significant increase of naked mtDNA (white arrows) in the cytoplasm of OHCs. MT treatment partially recovered the TFAM-mtDNA interaction and significantly alleviated the increase of naked mtDNA in OHCs (Figures 8A,B). These data indicated that the mtDNA-binding activity of TFAM was weakened in the cochlea following acoustic trauma. MT treatment could restore the binding function of TFAM.

## Discussion

MT is a novelly developed mitochondria-target antioxidant with the ability to pass through the phospholipid bilayer and accumulate in mitochondria. In the present study, we investigated the protective effect of MT against NIHL and the underlying mechanism in a rat model. We found that MT reduced noise-induced threshold shift and prevented OHC loss, IHC ribbon synapse loss, and ANF degeneration after the noise exposure. We demonstrated that MT exerted its protective effect on hearing by reducing the level of oxidative stress and mtDNA damage, restoring TFAM-mtDNA interaction and mitochondrial function of the inner ear.

HCs and SGNs are two important cells responsible for the perception, mechanoelectrical transduction, and transmission of auditory signals. We found that acoustic trauma led to OHC loss while IHC remained intact. This differential vulnerability may be due to the difference in intrinsic antioxidant capacity between IHCs and OHCs (Sha et al., 2001; Rosenhall et al., 2019). However, the amount of paired ribbon synapses between IHCs and SGNs was significantly reduced after the noise exposure. ANFs are the peripheral neurites of SGNs. Noise-induced ANF degeneration was observed in our experiments. In addition, the damage of cochlear ribbon synapses and ANFs is also the pathologic change of noise-induced hidden hearing loss (NIHHL; Kujawa and Liberman, 2009; Liberman and Kujawa, 2017). The protective effect of MT on ribbon synapses and ANFs suggested its promising application to prevent NIHHL, which needs further investigations.

The present study showed increased oxidative stress indicated by cochlear 4-HNE and DHE level, which is consistent with previous studies (Park et al., 2014; Paciello et al., 2020; He et al., 2021b). We observed increased mtDNA oxidative damage indicated by 8-OHdG in SGNs 1 h after noise exposure. The cochlear mitochondrial function indicated by the ATP content was also impaired 1 h after the noise exposure, which is consistent with a previous study (Chen et al., 2012). Maintaining an adequate content of mtDNA copy number is important for cellular energy metabolism. Mitochondrial ROS induced by noise caused oxidative damage to mtDNA, resulting in mitochondrial dysfunction and energy insufficiency. SGNs are auditory neurons and consume a lot of energy in axoplasmic transport and electrical signal transmission. When the number and function of the mitochondria in SGNs are impaired, the transport of cellular cargos including neurotrophin might be disrupted, resulting in the degeneration of the neurites of SGNs. Mitochondrial biogenesis is an activity to generate new mitochondria from the existing ones. This process requires the coordination of mtDNA replication and expression and is regulated by several transcription factors. The present study showed that mtDNA content and expression level were both decreased after noise exposure, which indicated the impaired mitochondrial biogenesis in the inner ear after the acoustic trauma. Yu and colleagues found mtDNA common deletion induced by D-gal increased susceptibility to NIHL in rats (Yu et al., 2014), suggesting the correlation between mtDNA integrity and the pathogenesis of NIHL. Interestingly, we found that the mtDNA expression level decreased immediately following the acoustic trauma (1 h after the noise exposure). In contrast, mtDNA copy number did not reduce until the third day after the noise exposure. The possible explanation is that DNA is more stable and has a longer half-life than RNA. It takes time for mtDNA to be degraded after oxidative stress damage. Previous studies also showed that mtDNA copy numbers decrease several days after the environmental stress (Bagul et al., 2018; Sugasawa et al., 2021).

The PGC-1α/NRF-1/TFAM axis is one of the most important pathways in regulating mitochondrial biogenesis. However, there are few studies on the alteration of the PGC-1α/NRF-1/TFAM pathway during acoustic trauma. In order to further explore the mechanism by which mtDNA content and expression are impaired in NIHL, we analyzed the expression level of this pathway. We found that the expression level of TFAM significantly decreased after the noise exposure, while the expression level of PGC-1α and NRF-1 remained unchanged. In addition, the TFAM function, as indicated by the mtDNA-binding ability, was also disrupted in OHCs after the noise exposure. These results indicate that noise-induced ROS specifically impairs the expression and function of TFAM without affecting the upstream PGC-1α and NRF-1. OHCs exhibit electromotility and mechanically amplify sound-evoked vibrations, which is an energy-consuming process. The impaired TFAM-mtDNA interaction leads to an increase of naked mtDNA molecules, which are vulnerable to oxidative damage by ROS. The abnormal mtDNA hinders the expression of mitochondrial respiratory chain proteins and ATP synthesis and might contribute to OHC dysfunction and death. A previous study showed that the phosphorylation and acetylation of TFAM within its HMG-box 1 domain reduced the DNA binding affinity, leading to TFAM-mtDNA disassembly (King et al., 2018). In addition, phosphorylation at serine 55/56 facilitated rapid degradation of TFAM by the Lon protease in mitochondria (Lu et al., 2013). Since it has been reported that acoustic trauma activates the protein kinase such as AMPK and extracellular signal-regulated protein kinase (ERK) in the inner ear (Kurioka et al., 2015; Hill et al., 2016), phosphorylation regulation might play a relevant role in the impaired expression and binding function of TFAM after the noise exposure. This speculation deserves further exploration in future studies.

Since oxidative stress plays an important role in the pathogenesis of NIHL, the antioxidants have been extensively studied in attenuating NIHL (Pak et al., 2020). A number of antioxidants show protective effects against NIHL in animal models such as glutathione, N-acetylcysteine (NAC), D-methionine, vitamin C, water-soluble coenzyme Q10, ebselen, resveratrol, and HK-2 (Ohinata et al., 2000; Seidman, 2003; Duan et al., 2004; Lynch et al., 2004; McFadden et al., 2005; Fetoni et al., 2009; Ewert et al., 2012; Chen et al., 2020), some of which have entered clinical trials (Kopke et al., 2015; Kil et al., 2017; Rosenhall et al., 2019). However, conventional antioxidants cannot remove only excess ROS without suppressing physiological ROS which are important for signal transduction. Recently, mitochondria-target antioxidants have been developed for the treatment of oxidative stress-related disease for their capacity to scavenge ROS from the source (Fujimoto and Yamasoba, 2019). MT is the antioxidant TEMPOL conjugated to the lipophilic triphenylphosphonium (TPP) cation, which can easily pass through the phospholipid bilayer and accumulate several hundred-fold into mitochondria (Trnka et al., 2008). A previous study shows that MT is BBB-penetrating and can be used as the contrast media in enhanced-magnetic resonance imaging (MRI) of the brain (Zhelev et al., 2013). In the present study, we demonstrated the existent of MT in the inner ear after the systemic administration. The lipophilic property of MT makes it easy to pass through the BLB and exert the antioxidant effects in the inner ear. MT exhibited a protective effect on hearing and cochlear cells against acoustic trauma. MT treatment reduced mitochondrial ROS production induced by noise exposure and therefore alleviated mtDNA oxidative damage and mitochondrial dysfunction. These results are consistent with a recent study (Zhao et al., 2021) which showed that MT alleviated ischemic acute kidney injury via reducing mitochondrial ROS and promoting TFAM-mediated mtDNA maintenance.

This study has several limitations. First, the methods used for hearing function evaluation were relatively simple. Four tested frequencies of the ABR covered about 50% length of the cochlea (from 30% to 80%), and may not represent well the hearing status of the animal. In future studies, we will use distortion product otoacoustic emissions (DPOAE) and compound action potential (CAP) for a comprehensive assessment of hearing function. Secondly, due to the technical difficulties, the RNA of HCs and SGNs was unable to be separately isolated. The alterations of mRNA expression of HCs and SGNs could not be analyzed respectively. Other methods such as RNA fluorescence *in situ* hybridization (FISH) will be used in our future studies. Finally, electron microscopy analysis of mitochondria was not performed in the present studies. Since mitochondrial fusion and fission are crucial processes to maintain mitochondrial homeostasis (Chan, 2020), the morphological changes of cochlear mitochondria in NIHL will be further studied in the following work.

In summary, our results first show TFAM reduction and the disruption of TFAM-mtDNA interaction in NIHL. Noise-induced ROS lead to mtDNA oxidative damage, impairing mtDNA expression and mitochondrial biogenesis. MT treatment exhibits a protective effect on hearing, OHCs, IHC synapses, and ANFs against acoustic trauma partially by maintaining TFAM-mtDNA interaction and mitochondrial biogenesis based on its strong capacity of mitochondrial ROS scavenging.

## Data Availability Statement

The original contributions presented in the study are included in the article/Supplementary Material, further inquiries can be directed to the corresponding author/s.

## Ethics Statement

The animal study was reviewed and approved by Institutional Animal Care and Use Committee of Fourth Military Medical University.

## Author Contributions

L-JL and QY designed the research. J-WC, P-WM, HY, W-LW, and P-HL performed the experiments. X-RD, Y-QL, and L-JL analyzed the data. J-WC prepared the figures. J-WC, QY, and L-JL wrote the manuscript. All authors contributed to the article and approved the submitted version.

## Conflict of Interest

The authors declare that the research was conducted in the absence of any commercial or financial relationships that could be construed as a potential conflict of interest.

## Publisher’s Note

All claims expressed in this article are solely those of the authors and do not necessarily represent those of their affiliated organizations, or those of the publisher, the editors and the reviewers. Any product that may be evaluated in this article, or claim that may be made by its manufacturer, is not guaranteed or endorsed by the publisher.
