# Supplementary material for: mito-TEMPO Attenuates Oxidative Stress and Mitochondrial Dysfunction in Noise-Induced Hearing Loss via Maintaining TFAM-mtDNA Interaction and Mitochondrial Biogenesis
Source: Front Cell Neurosci. 2022 Feb 8;16:803718. doi: 10.3389/fncel.2022.803718 (PMC8861273; doi:10.3389/fncel.2022.803718)
Supplement: Supplementary file 1 [file Data_Sheet_1.docx]

# Supplementary Material


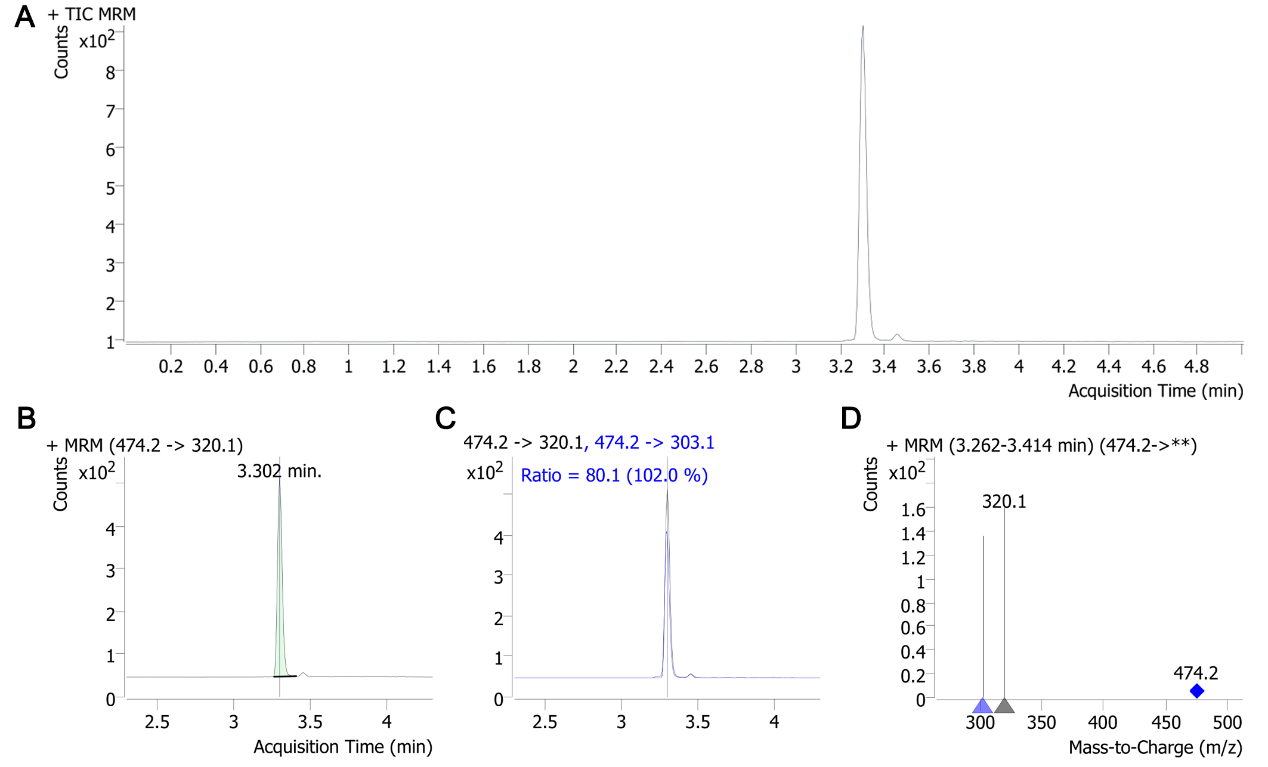


**Supplementary Figure 1. The** **chromatogram and** **mass spectrum of the** **perilymph sample 15 min after the administration of mito-TEMPO.** (A) The total ion current (TIC) chromatogram of the perilymph sample. (B and C) The extracted ion chromatograms (EIC) of two daughter ions (m/z = 320.1 and 303.1). (D) The tandem mass spectrum of m/z 474.2 from the perilymph sample.


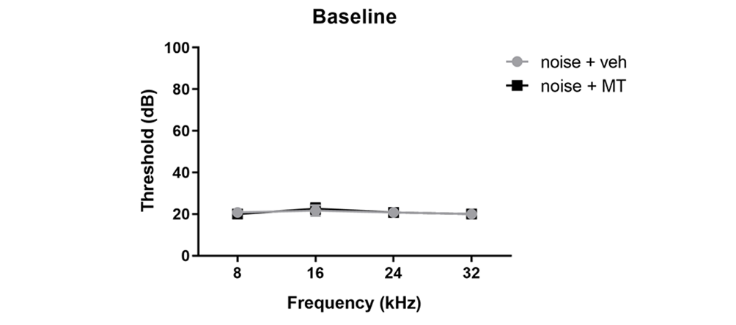


**Supplementary Figure 2.** **The baseline auditory threshold of the animals.** The baseline auditory thresholds of the left ear of animals over 8-32 kHz evaluated via ABR testing. Data are presented as means ± SD, n = 6 for each group.


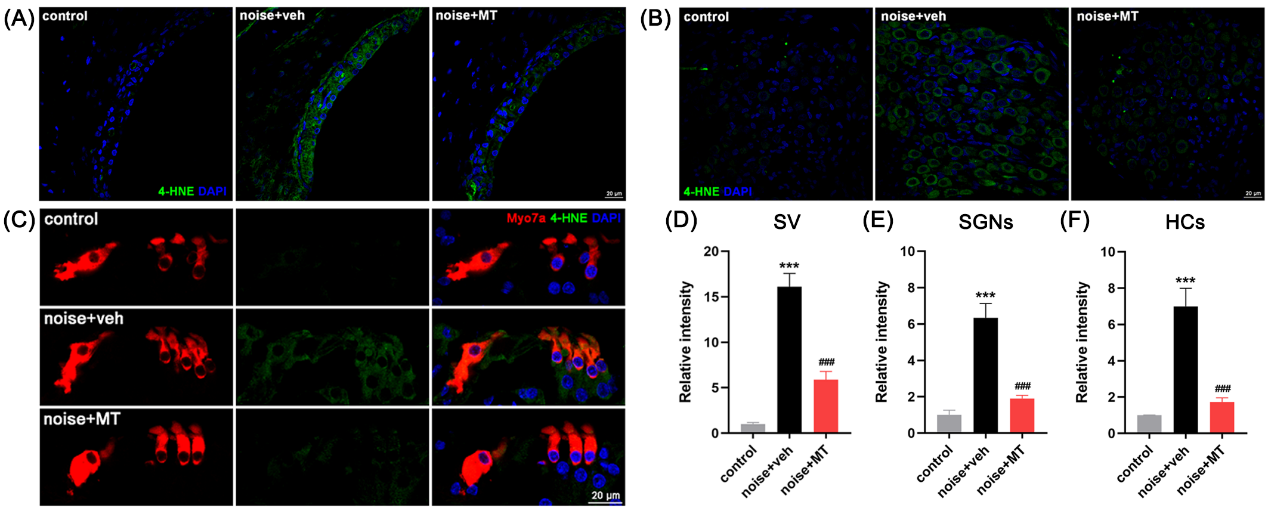


**Supplementary Figure 3. MT treatment mitigated noise-induced** **lipid peroxidation in the cochlea.** Representative images of 4-HNE (green), Myosin Ⅶa (red) and DAPI (blue) fluorescent staining in SV (A), SGNs (B) and HCs (C) of the base turn area of the midmodiolar sections 1h after the noise exposure. (D-F) Semi-quantitative analyses of the fluorescent intensity of 4-HNE in SV, SGNs and HCs 1h after the noise exposure. Data are presented as means ± SD, n = 4 for each group, ****p* < 0.001 vs control group, ###*p* < 0.001 vs vehicle-treated group. SV, stria vascularis; SGNs, spiral ganglion neurons; hair cells, HCs.
